# PP71 Mapping Current Decision-Making Pathways And Reimbursement Processes For High-Risk Devices In The European Union, European Economic Area, And UK: Scoping Review

**DOI:** 10.1017/S0266462325102894

**Published:** 2025-12-29

**Authors:** Rasha Alshaikh, Kieran Walsh, Fatma El-Komy, Susan Spillane, Marie Carrigan, Louise Larkin, Patricia Harrington, Shelley O’Neill, Conor Teljeur, Máirín Ryan, Caitriona O’Driscoll

## Abstract

**Introduction:**

Implementation of the Health Technology Assessment (HTA) Regulation (HTAR) marks a shift in the assessment of high-risk medical devices (MDs) and in vitro diagnostics (IVDs) across the European Union (EU) and European Economic Area (EEA). Understanding current decision-making pathways for reimbursement of these technologies is crucial to inform HTAR integration within national frameworks. This review aimed to understand how comparative safety and effectiveness evidence is used in reimbursement decisions.

**Methods:**

A scoping review was conducted in accordance with a registered protocol (osf.io/65bdk). Relevant documents were identified through searches of websites of HTA bodies and national health authorities across the EU, EEA, and UK. Relevant publications were identified from key databases (MEDLINE Complete, Embase, and the Cochrane Library) and supplemented by a comprehensive gray literature search. Data were extracted and checked by a second reviewer. National health officials further validated the extracted data.

**Results:**

The searches of official websites resulted in the retrieval of 369 relevant records. Database and gray literature searches resulted in the retrieval of 953 records, with 134 of these being included after full-text screening. The results revealed diverse reimbursement pathways and HTA processes across the EU, EEA, and UK for high-risk devices. Findings highlighted the variability in reimbursement processes. Some countries rely on centralized decision-making bodies, while others rely on regional or hospital-specific approaches. The findings also emphasized the limited integration of comparative safety and effectiveness data in the reimbursement frameworks of some countries and underscored the complexity of reimbursement processes across the European region.

**Conclusions:**

This review identified key variations in reimbursement pathways for high-risk MDs and IVDs across the EU, EEA, and UK. The review of the European reimbursement decision-making landscape and the role of HTA underscores the importance of sharing experiences to facilitate the efficient use of HTAR-generated evidence in decision-making and to support unified patient access to safe and effective technologies.

